# ﻿Temporal and spatial herbarium density of *Favratiazoysii* (Wulfen) Feer (Campanulaceae): Insights into type material and historical distribution of an alpine endemic

**DOI:** 10.3897/phytokeys.257.147262

**Published:** 2025-06-13

**Authors:** Yasaman Ranjbaran, Marco Canella, Andreas Fleischmann, Nora Codato, Valentina Boscariol, Špela Pungaršek, Sara Natale, Francesco Dal Grande

**Affiliations:** 1 Department of Biology, University of Padova, Viale G. Colombo 3, 35131 Padova, Italy University of Padova Padova Italy; 2 Botanical Garden of Padova, 35123 Via Orto Botanico 15, Padova, Italy Botanical Garden of Padova Padova Italy; 3 National Biodiversity Future Center, Palermo, Italy National Biodiversity Future Center Palermo Italy; 4 Botanische Staatssammlung München, Menzinger Str. 67, 80638 München, Germany Botanische Staatssammlung München München Germany; 5 Department of Earth and Marine Sciences, University of Palermo, Via Archirafi 22, 90123 Palermo, Italy University of Palermo Palermo Italy; 6 Slovenian Museum of Natural History, Prešernova cesta 20, 1000 Ljubljana, Slovenia Slovenian Museum of Natural History Ljubljana Slovenia

**Keywords:** Alpine flora, Campanula, chasmophytes, herbaria, typification

## Abstract

*Favratiazoysii* (Wulfen) Feer is a chasmophyte endemic to the south-eastern Alps and the sole representative of the genus *Favratia* Feer. Its primary occurrences are in Julian Alps and on Mt. Storžič (Kamnik-Savinja Alps) in Slovenia. This study aims at elucidating the original material utilised by Wulfen in the initial description of *Campanulazoysii*, later reclassified as *F.zoysii* by Feer, while also exploring the temporal and spatial density of the species’ herbarium collections. To this end, we examined preserved collections from six institutions, including botanical gardens and museums. A total of 127 herbarium vouchers were analysed to identify the specimens referenced by Wulfen. This comprehensive examination of historical collections, spanning over two centuries, allowed us to designate a lectotype, thereby providing a clear typification of *F.zoysii*. Additionally, we analysed the frequency and geographic distribution of herbarium specimens, a concept referred to as temporal and spatial herbarium density. This combined typification and spatial-temporal analysis not only strengthens the taxonomic clarity of the species, but also provides valuable insights into its botanical history, distribution patterns and conservation needs.

## ﻿Introduction

*Favratiazoysii* (Wulfen) Feer is an alpine chasmophytic species endemic to Slovenia, Austria and Italy, with a distribution limited to the south-eastern Alps. The species’ primary populations are concentrated in the Triglav Massif and the Kamnik-Savinja Alps in Slovenia. While the range extends further across the Julian Alps (encompassing Slovenia and Italy), the Karavanke Mountains (shared between Slovenia and Austria), the northern border of the Trnovo Forest Plateau (locally referred to as Trnovski gozd) and the Carnic Alps (Cohrs 1954; Hartl et al. 1992; Jogan et al. 2001; Aeschimann et al. 2004; Dakskobler and Praprotnik 2004; Dakskobler and Strgar 2017; Bartolucci et al. 2018; Pignatti et al. 2017; GBIF (2024a, b); Triglav National Park ranger service, personal communication). *Favratiazoysii* is predominantly found in crevices in limestone and dolomite rock formations, though it is occasionally observed on gravel and scree. It is associated with the *Potentilloclusianae*-*Campanuletumzoysii* vegetational community and typically thrives at elevations ranging from 1800 to 2400 m a.s.l. (Valič 2006; Hirtl 2016; Pignatti et al. 2017). However, an isolated occurrence is documented at lower altitudes between 1000 to 1050 m a.s.l. in Govci (Trnovo Forest Plateau, Slovenia; Dakskobler (2004)).

*F.zoysii* has captivated botanical interest since its discovery. It was described as *Campanulazoysii* Wulfen by Franz Xaver von Wulfen (1728–1805) (Wulfen 1789), a self-taught naturalist and influential botanist (Klemun 1989). He established a key partnership with Karl von Zois (1756–1799), a Carniolan baron who first provided Wulfen with specimens and site details of *F.zoysii* (Praprotnik 1991, 2015). In 1890, Swiss botanist Heinrich Feer reclassified the species as *Favratiazoysii*, citing two distinctive morphological traits: lesiniform teeth measuring 5 mm and a cylindrical corolla tapering at the apex (Feer 1890). Today, *F.zoysii* holds a prominent position within taxonomic, conservation and biodiversity frameworks, with its inclusion in major international databases, such as World Plants, World Flora Online, Plants of the World Online, GBIF and the IUCN Red List.

Although classified as “Least Concern” by the IUCN Red List due to the demographic stability of its populations, *F.zoysii* occupies a relatively small area, with its area of occupancy estimated at less than 500 km^2^ (IUCN 2024). The species is listed in Annexes II and IV of the European Habitats Directive (EEC 1992a, b), requiring regular assessments of its conservation status and designation of priority conservation areas. Currently, *F.zoysii* is recorded in five Natura 2000 sites in Italy, five in Slovenia and four in Austria.

Although populations of *F.zoysii* are generally stable, the species’ restricted distribution makes it particularly susceptible to environmental changes. Habitat disruption from climate change and land-use modifications pose significant threats. Specific risks include overgrazing by sheep at high altitudes and increased competition with other herbaceous species driven by the “greening” of mountain peaks (Porro et al. 2021). Furthermore, genetic drift may occur due to population isolation, although no population genomic studies on this species have been conducted to date.

Given the ecological significance of *F.zoysii*, it is striking that the species has not yet been typified. This study addresses that gap as part of a broader initiative aimed at typifying vascular plants described in the Italian Alps (e.g. Orsenigo and Galasso (2019); Orsenigo et al. (2019); Canella et al. (2024)). Establishing a specimen-based reference through the identification and documentation of type material is essential for maintaining taxonomic clarity and stability (Rapini 2014). Specifically, the objectives of this study were twofold: i) to assess the status of preserved collections of *F.zoysii*, including the number, age and distribution of vouchers over time and space; and ii) to designate the appropriate type material for this species. By incorporating an evaluation of temporal and spatial herbarium density, we not only addressed typification of *F.zoysii*, but also provided a broader framework for understanding how the species’ historical collections can provide information for its current distribution and conservation status.

## ﻿Materials and methods

### ﻿Species description and diagnostical traits

As a first step, diagnostic traits of the species were established, based on literature sources (Pignatti et al. 2017) to ensure a clear identification in both preserved collections and field observations. *F.zoysii* is a perennial herb, typically 5–10 cm in height, with a densely bushy and sub-glabrous form. The stems are ascending or erect and the leaves are entire and slightly fleshy. Basal leaves are oblanceolate-spatulate, whereas cauline leaves are smaller and range from lanceolate to linear. Flowers are one or few per stem and their orientation is either inclined or pendulous. The floral peduncles measure 5–10 mm in length, with a 5 mm calyx tube and equally long, awl-shaped teeth. The corolla, which is cylindrical and slightly constricted at the throat, measures 15–20 mm wide and bears obtuse teeth. The capsule is subspherical, angular and dehisces through pores located beneath the calyx teeth at the apex (Martini 1989). Hybridisation with *Campanulacochleariifolia* has been reported, resulting in individuals with intermediate traits (Ravnik 1967).

### ﻿Preserved specimens and living occurrences

We accessed the Global Biodiversity Information Facility (GBIF) (2024a, b) to obtain an overview of both living occurrences and preserved specimens of the study species. Search queries included the accepted name (*F.zoysii*) and its synonym (*C.zoysii*). To create a comprehensive reference database, we employed a mixed-method approach. Starting with GBIF data (GBIF 2024a), we incorporated additional sources; i) preserved specimens obtained through online consultations of non-GBIF-indexed collections and direct communication with herbarium curators, ii) additional living occurrences from Triglav National Park authorities and local botanists. This approach allowed us to compile a comprehensive list of specimens, documenting key details such as the collector, date of collection and locality.

### ﻿Typification

The typification process adhered to McNeill (2014) and Turland et al. (2018). Relevant literature sources were viewed, with particular emphasis on the protologue and the biography of Franz Xaver von Wulfen. We prioritised specimens with Wulfen as collector. For six institutions, we physically examined the samples, for a total of 127 herbarium vouchers.

## ﻿Results

### ﻿Living occurrences and preserved specimens

The GBIF living occurrences and human observations includes 131 georeferenced records. We added 138 occurrences, based on personal communications with local botanists and 22 entries provided by the Triglav National Park ranger service. This resulted in a comprehensive database comprising 291 georeferenced locations (Suppl. material 1).

The GBIF dataset of preserved specimens contains 350 herbarium vouchers (GBIF 2024b), with 41 deposited under the name *F.zoysii* and 299 specimens under *C.zoysii*. We supplemented this dataset with 113 additional entries: two from Botanical Garden of Padova (PAD), three from MUSE - Museo delle Scienze (TR), 16 from Slovenian Museum of Natural History (LJM) and 57 from Italian Central Herbarium (FI), one from Meise Herbarium (ME) and 34 from Botanische Staatssammlung Munchen (BSM), Suppl. material 1.

**Figure 1.**
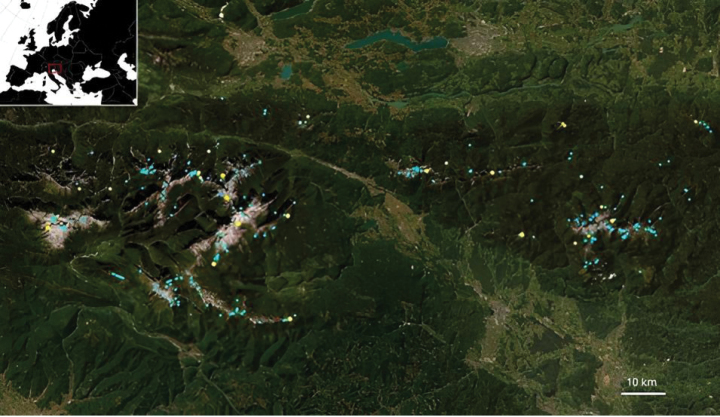
Map showing the current distribution of *F.zoysii* (light blue dots) and the provenance of preserved collection specimens with known collecting location (yellow dots).

The resulting distribution of current living occurrences and the original sampling locations for the herbarium vouchers is shown in Fig. 1. The collection location of herbarium vouchers clusters in 25 main locations, corresponding to mountain tops and ridges where *C.zoysii* matches its usual growing environment.

**Figure 2.**
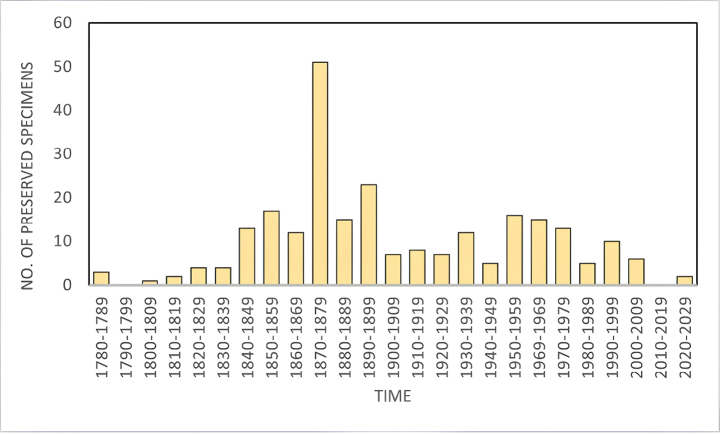
Temporal herbarium density. The number of herbarium specimens found is reported in a timeline. Columns represent the sum of herbarium specimens collected for each decade.

In our database, 251 specimens have a recorded year of collection. The oldest specimen dates to 1788, while the most recent was collected in 2022, spanning a period of more than two centuries (Fig. 2). On average, 10.04 ± 10.7 herbarium vouchers were collected per decade between 1780 and the present. The timeline reveals a notable concentration of specimens collected during the second half of the 19^th^ century, which accounts for 47% of the total database of preserved specimens. The peak collection period occurred during the decade of 1870–1879 with 51 specimens.

### ﻿Typification and nomenclatural notes

A total of 127 herbarium specimens were physically examined across six institutions, all containing promising candidates for typification (Table 1).

**Table 1.** List of Herbaria for which the specimens have been physically examined.

Herbarium name	Herbarium code	Location	Number of *F.zoysii* specimens
Botanische Staatssammlung Munchen	BSM	Munich (Germany)	34
Botanical Garden of Padova	PAD	Padua (Italy)	2
MUSE - Museo delle Scienze	TR	Trento (Italy)	3
Meise Herbarium	ME	Meise (Belgium)	17
Slovenian Museum of Natural History	LJM	Ljubljana (Slovenia)	16
Italian Central Herbarium	FI	Florence (Italy)	55
Total Herbarium vouchers			127

Amongst these, 91 specimens included notes identifying the collector. Due to the small size of the plants, many herbarium sheets contained multiple individuals, resulting in a total of 251 individuals. The typification of *Favratiazoysii* (Wulfen) Feer has been formally defined as follows:

***Favratiazoysii* (Wulfen) Feer, Bot. Jahrb. Syst. 12(5): 610 (1890)**

#### Type.

**Figure 3.**
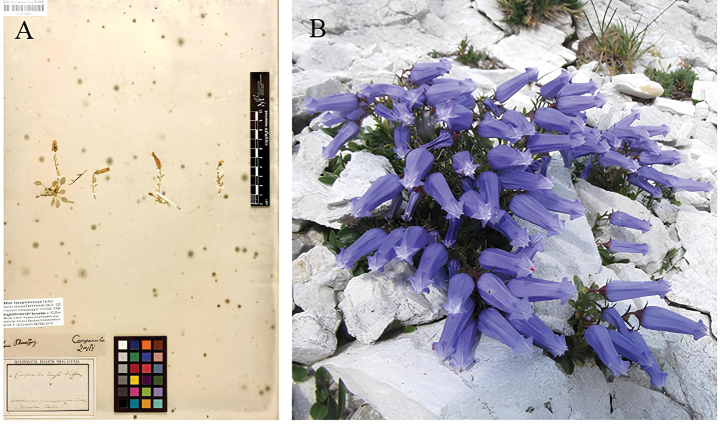
Photo of the newly-designated lectotype, photo taken by Y. Ranjbaran (**A**) and plant at the *locus classicus* in Mount Storžič, Slovenia, western path to the mountain top, photo taken by Š. Pungaršek (**B**).

“vom Storchez / in summitate montis Storchez” referring to the current locality Mount Storžič (46.3502353 N, 14.4047511 E, WGS 84). F.X. von Wulfen.lectotype designated here: M-0214994 / 629961 / 287462 3, (Fig. 3A) image available at https://www.gbif.org/occurrence/1848896556.

The current occurrence of the species has been confirmed in the locus classicus during fieldworks in summer 2023 (Fig. 3B).

This extensive collection history provides an invaluable dataset for analysing changes in species distribution and broader ecological trends over time.

The temporal distribution of herbarium vouchers reveals a peak in collections between 1830 and 1880, a half-century that serves as a critical reservoir of metadata for historical studies. This rich repository of specimens forms a robust foundation for future research, particularly in investigating the impacts of climatic and anthropogenic changes on the species habitat.

## ﻿Discussion

### ﻿Number of samples and GBIF representativeness

GBIF data have proven to be a useful initial source of information regarding the current distribution of *F.zoysii*. The clear diagnostic characteristics of this species make it easily recognisable, with a very low chance to misidentification during the flowering period. This facilitates the validation of human observations from amateur botanists, mountaineers and plants enthusiasts. Consequently, occurrences from citizen-science initiatives, such as INaturalist, accessible via GBIF, represent an additional valuable source of data. However, we also have shown that GBIF data can be easily fostered through a network of collaborations though non-public repositories. In our case, we more than doubled the number of georeferenced locations through data requests to local botanists and a National Park authority. Similarly, we found that many herbaria still hold non-indexed herbarium vouchers. Direct requests to curators remain a valuable source of information on preserved collections. A recent study on 130 species of the genus *Campanula* (including *F.zoysii* as *C.zoysii*) highlighted that widely distributed species, such as *C.rotundifolia* and *C.rapunculoides*, are often the most common in preserved collections (Dincă and Vechiu 2020). However, the same study also emphasised that some narrowly-distributed endemics are well-represented in herbarium collections. This appears to be the case for *F.zoysii*, for which we found abundant and well-distributed records in preserved collections, both in terms of spatial and temporal distribution.

### ﻿Typification

Type material was selected through a dataset of 461 herbarium vouchers with 128 physically examined. The selected voucher encloses two entire flowering individuals and two additional flowering ramets. All four entities are well preserved, with the diagnostic characters of the species clearly visible and easily recognisable. The newly-designated lectotype is stored at the Botanische Staatssammlung Munchen (BSM) and the scan of the voucher can be consulted via GBIF. The *locus classicus* has been shown to be abundantly populated by *F.zoysii*. The population found on Mount Storžič includes both flowering and seedling individuals, in contrast to many other locations visited during fieldwork in Triglav complex, Grintovec and Karavanke (unpublished data).

### ﻿Spatial-temporal distribution of *Favratiazoysii*

Regarding the distribution of *F.zoysii*, our study shows that all historical locations are supported by current occurrences. However, some modern populations, such as those in the Krn and Mahavšček complex in Slovenia, Eastern Karavanke in Austria, and Western Carnia in Italy, are under-represented in the preserved specimens. In contrast, the Triglav complex has been abundantly represented. The herbarium vouchers are concentrated in a limited number of major peaks. This clustering can be explained by the species’ highly-localised growing environment, which occurs almost exclusively on exposed cliffs near summits. Consequently, many of the georeferenced points overlap, forming clusters around the summits. Despite these geographical clustering, the herbarium vouchers span the full east-west and north-south distribution range of *F.zoysii*, covering the species’ entire natural range. The long collection history of living collections offers an invaluable dataset for examining changes in species distribution and other trends over time. The temporal distribution of the herbarium vouchers reveals it to be a valuable reservoir of plant material and metadata for historical and genetic studies of material, with a peak in collections between 1830 and 1880. This wealth of material presents a strong foundation for future research, particularly in understanding how the population genetics of this species have changed. In fact, the recent “Renaissance of herbaria” (Burbano and Gutaker 2023), fuelled by advances in ancient DNA genomics (Bieker and Martin 2018), has highlighted the critical role of herbarium specimens not only for typification and taxonomy, but also as invaluable tools for tracking biodiversity through space and time. These collections provide unique insights into the impacts of environmental changes on both economically important crops and wild species. *F.zoysii* is an ideal candidate for such herbariomic studies due to its extensive temporal and spatial herbarium coverage, with specimens collected over more than two centuries across its distribution which covers the current range and make the comparison between past and present populations possible. This wealth of material, along with detailed metadata (e.g. collector information, GPS coordinates and habitat details), offers a rich resource for high-throughput sequencing, enabling the exploration of genetic variability across time and space. By comparing historical specimens with fresh samples, it is possible to analyse demographic trends and shifts in genetic diversity. Such analyses are crucial for understanding genetic erosion over time (Díez-del-Molino et al. 2018), particularly in species with long collection histories. The evaluation of temporal and spatial herbarium density, as conducted in this study, is rarely applied, yet essential for identifying priority species for herbariomics research.

## ﻿Conclusions

This study highlights the importance of herbarium collections not only for typification, but also as invaluable tools for assessing the long-term effects of environmental changes on species distribution. The metadata associated with these specimens, such as collection date, locality and habitat details, are crucial for evaluating the historical distribution of *F.zoysii* and determining whether shifts in elevational range or population size have occurred over time. By combining historical herbarium specimens with modern field data, researchers can assess potential extinctions or uncover areas where the species might have shifted its range. In addition, the comprehensive temporal and spatial dataset assembled here provides a foundation for future herbariomics research, enabling the exploration of genetic variability across centuries.

## ﻿Additional information

### Author ORCIDs

Yasaman Ranjbaran https://orcid.org/0000-0002-4682-4270

Marco Canella https://orcid.org/0000-0002-8548-4109

Andreas Fleischmann https://orcid.org/0000-0002-4917-4736

Nora Codato https://orcid.org/0009-0006-3976-6072

Valentina Boscariol https://orcid.org/0009-0005-4248-2364

Špela Pungaršek https://orcid.org/0009-0001-7783-2134

Sara Natale https://orcid.org/0000-0003-4406-4252

Francesco Dal Grande https://orcid.org/0000-0002-1865-6281

### Data availability

All of the data that support the findings of this study are available in the main text or Supplementary Information.

## Supplementary materials

Supplementary material 1 Supplementary data Data type xlsx Explanation note **table S1_a.** Dataset of the living occurrences with geographic coordinates of *Favratiazoysii* Wulfen. CountryCode and institutionCode are according GBIF.**table S1_b.** Dataset of the preserved specimens of *Favratiazoysii* Wulfen. CountryCode and institutionCode are according GBIF. https://binary.pensoft.net/file/1352609This dataset is made available under the Open Database License (http://opendatacommons.org/licenses/odbl/1.0/). The Open Database License (ODbL) is a license agreement intended to allow users to freely share, modify, and use this Dataset while maintaining this same freedom for others, provided that the original source and author(s) are credited.Yasaman Ranjbaran, Marco Canella, Andreas Fleischmann, Nora Codato, Valentina Boscariol, Špela Pungaršek, Sara Natale, Francesco Dal Grande
